# Laparoscopic approach for very large benign ovarian cyst in young woman

**DOI:** 10.4103/0972-9941.58502

**Published:** 2009

**Authors:** Fulvio Tagliabue, Paola Acquaro, Gianmaria Confalonieri, Salvatore Spagnolo, Antonio Romelli, Melchiorre Costa

**Affiliations:** Department of Surgery, ‘A. Manzoni’ Hospital, Via dell'Eremo 9/11, 23900 Lecco, Italy

**Keywords:** Benign ovarian cyst, laparoscopy, ovary

## Abstract

Ovarian cysts are the most common cause of pelvic masses in women, and in the majority of the cases, women are in their fertile age. Today, the surgical treatment has become more conservative and less invasive; hence, a laparoscopic approach in the presence of benign cysts has become a gold standard. Herein, we report a case of a 21-year-old woman referred to our Surgical Department for an abdominal mass, discovered with a computerised tomographic scan, of 20 ×10 × 25 cm arising from the left ovary, treated with the laparoscopic approach.

## INTRODUCTION

Ovarian cysts are most common cause of pelvic masses in women, and in the majority of the cases are women in fertal age. Today the surgical treatment has become more conservative and less invasive, hence laparoscopic approach in presence of benign cysts has become a gold standard.

## CASE REPORT

**Figure 1:**
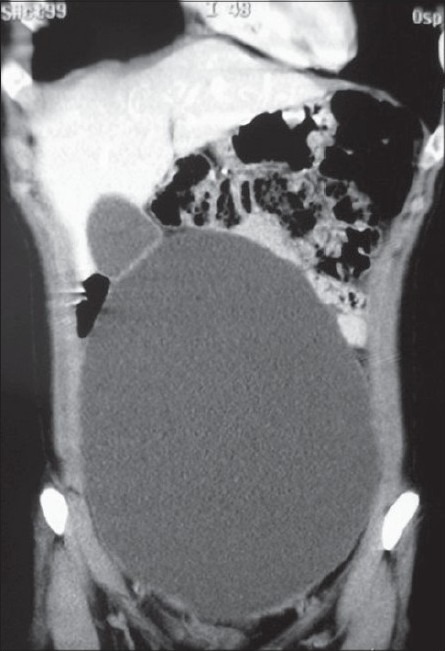
CT scan image of the large cyst

A 21-year-old nulligravid woman was seen for the first time at our department after 8 months of progressive abdominal distension without other symptoms. She did not have a family history for cancer. She referred no bowel or urinary disturbance and her periods were regular. On examination, the abdomen was distended by a large abdominal mass that reached above the level of the umbilicus. Transvaginal ultrasonography did not show abnormalities. The abdominal CT scan showed a large simple cyst with a thin wall uniloculated measuring approximately 19 ×11 × 26 cm occupying the pelvis, ipo and mesogastrium [Figure 1].

**Figure 2:**
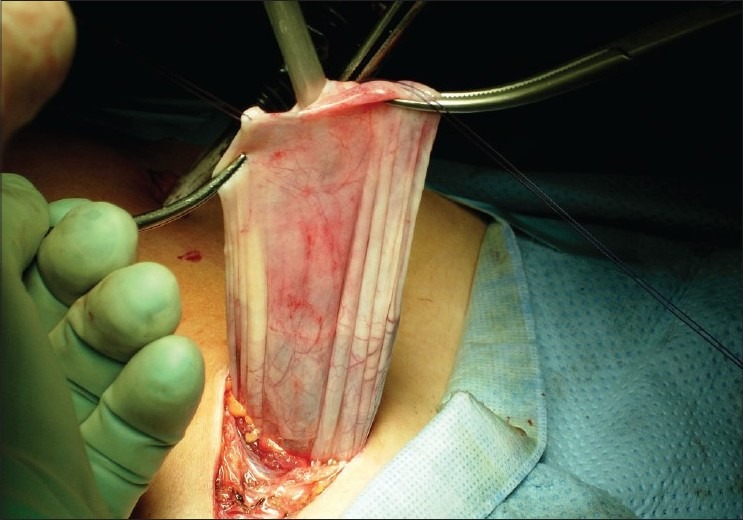
Extraction of the cyst through Pfannesteil incision with partial aspiration of the cyst

Her serum tumour markers were all within the normal range. The pre-operative evaluation revealed features of a benign cyst, and her general health conditions were good so the authors decided to use the laparoscopic approach. During the operation, the cyst was found to be arising from the left ovary and was about 20 ×10 × 25 cm in size. The rest of the abdominal cavity seemed normal [Figure 2]. The cyst had dense adhesions with the left ovary and had many venous vessels. It was impossible to separate the ovary from the cyst. Therefore, the cyst and the ovary were removed en bloc through a suprapubic incision near the hairline.

**Figure 3:**
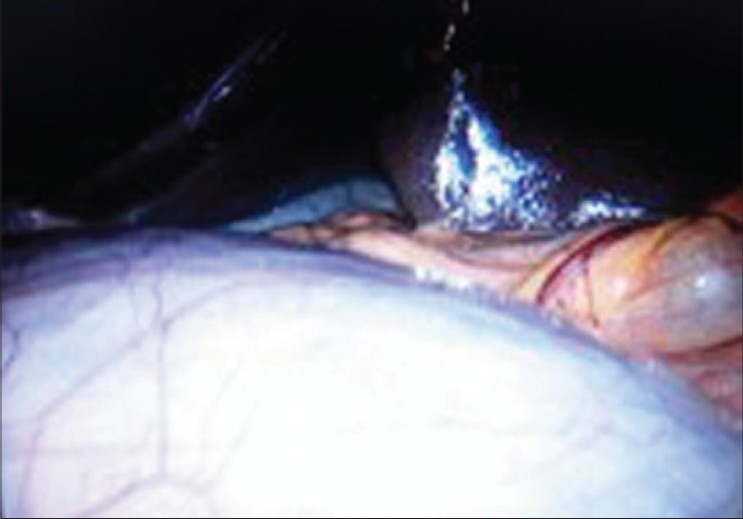
Intraoperative image shows the large cyst close to the stomach

Before removing the cyst, 4000 ml of clear liquid was aspirated and a sample was taken for the cytological study [Figure 3].

The pathological examination revealed a benign serous cystadenoma. The patient has been discharged 3 days later in good health.

## DISCUSSION

The employment of laparoscopy for the surgical management of benign ovarian cysts has become popular. Although it is a challenging task when the cysts are large. A randomized prospective study[1] comparing laparoscopy and laparotomy in the management of patients with benign ovarian masses less than 10 cm in diameter reported a significant reduction in operative morbidity, postoperative pain and analgesic requirement, hospital stay and recovery period. However, the same results can be obtained with large cysts also.

In the surgical treatment of benign ovarian cysts in young women, independently of its size, one of the main goals that all surgeons need keep in mind is to preserve the reproductive and hormonal functions of the ovaries and prevent recurrence. However, relatively frequent, this cannot be achieved because the cysts have strong adhesions with the ovary as reported in this paper.

The laparoscopic approach for the removal of cysts with a diameter greater than 10 cm presents different difficulties; the most important are as follows: First, the rupture of the cysts with spillage of its contents during the introduction of a trocar or Veress needle. The authors prefer an open technique with the use of a Hasson trocar near the umbilicus. They did not aspirate the cyst prior to the operation. A recent literature revealed that some authors prefer cyst size reduction prior to laparoscopy and it may be obtained using different techniques such as ultrasound-guided aspiration[2] or with the use of the Bonanno catheter.[3] Second, there is a limited visualization and work space that causes difficulties in identifying important structures such as the ureters. Third, the extraction of the cyst is not simple and in the presence of potential malignancy, spillage of the cyst contents could lead to dissemination and seeking where the trocars were placed. The significance of spillage in cases of malignancy is controversial. The concern about spillage of tumour cells and its possible worsening of prognosis arose from early studies of tumour rupture. Dembo *et al*.[4] studied the rate of relapse in 519 stage 1 epithelial ovarian cancer patients by logistic regression and multivariate analysis. The only factors that influence tumour relapse were tumour grade, the presence of dense adhesions or the presence of large volume ascites.[1] Therefore, the intra-operative rupture of tumour did not influence the prognosis. This statement is clearly supported by Sevelda *et al*.,[5] who studied the survival of patients with moderately and poorly differentiated stage 1 ovarian carcinoma and they concluded that there is no difference in the survival rate between the patients with intra-operative cyst rupture.

The authors believe that in selecting the patient one must consider both general health conditions and the morphology of the cysts with preoperative imaging indicating benign features of the cysts.
